# Interchangeability of the Wii Balance Board for Bipedal Balance Assessment

**DOI:** 10.2196/rehab.3832

**Published:** 2015-08-27

**Authors:** Bruno Bonnechère, Bart Jansen, Lubos Omelina, Marcel Rooze, Serge Van Sint Jan

**Affiliations:** ^1^Laboratory of Anatomy, Biomechanics and Organogenesis (LABO)Faculty of MedicineUniversité Libre de BruxellesBrusselsBelgium; ^2^Department of Electronics and InformaticsETROVrije Universiteit BrusselBrusselsBelgium; ^3^iMindsDepartment of Medical ITGhentBelgium; ^4^Institute of Computer Science and MathematicsSlovak University of TechnologyBratislavaSlovakia

**Keywords:** force plate, balance board, balance performance, validity, repeatability

## Abstract

**Background:**

Since 2010, an increasing interest in more portable and flexible hardware for balance and posture assessment led to previously published studies determining whether or not the Wii Balance Board could be used to assess balance and posture, both scientifically and clinically. However, no previous studies aimed at comparing results from different Wii Balance Boards for clinical balance evaluation exist.

**Objective:**

The objective of this crossover study is to assess the interchangeability of the Wii Balance Board.

**Methods:**

A total of 6 subjects participated in the study and their balance was assessed using 4 different Wii Balance Boards. Trials were recorded simultaneously with Wii Balance Boards and with a laboratory force plate. Nine relevant clinical parameters were derived from center of pressure displacement data obtained from Wii Balance Board and force plate systems. Intraclass correlation coefficients (ICC), *F* tests, and Friedman tests were computed to assess the agreement between trials and to compare the Wii Balance Board and force plate results.

**Results:**

Excellent correlations were found between the Wii Balance Board and force plate (mean ρ =.83). With the exception of 2 parameters, strong to excellent agreements were found for the 7 remaining parameters (ICC=.96). No significant differences were found between trials recorded with different Wii Balance Boards.

**Conclusions:**

Our results indicate that for most of the parameters analyzed, balance and posture assessed with one Wii Balance Board were statistically similar to results obtained from another. Furthermore, the good correlation between the Wii Balance Board and force plate results shows that Wii Balance Boards can be reliably used for scientific assessment using most of the parameters analyzed in this study. These results also suggest that the Wii Balance Board could be used in multicenter studies and therefore, would allow for the creation of larger populations for clinical studies.

**Trial Registration:**

Ethical Committee of the Erasme Hospital (CCB B406201215142).

## Introduction

The potential use of the Nintendo Wii Balance Board for assessing balance and posture has already been previously investigated [1]. A recent paper presented a comparison between a laboratory force plate and a Wii Balance Board to measure postural control [2]. The strength of this study was that by simultaneously recording with the force plate and the Wii Balance Board, subject variability was removed. Despite some doubts on the methodology and conclusions of the latter paper [3], it appears that the Wii Balance Board can be used to assess posture [4].

Most previously published papers on this particular topic reported a high correlation between a force plate and a Wii Balance Board for evaluating center of pressure trajectories. Such conclusions have therefore encouraged the use of Wii Balance Board hardware in daily clinical practice to assess balance in various pathological conditions such as Parkinson disease, orthopedics, and elderly assessment (eg, [5]). Many accuracy studies can be found in the literature (ie, comparing Wii Balance Board to some laboratory gold standard such as force plate hardware). Surprisingly, only one study can be found in the literature that assesses the repeatability of the measurements performed using different Wii Balance Board systems [6]. These authors compared force and localization of the center of pressure recorded with different Wii Balance Boards using different weight placed in various places on the Wii Balance Board. The authors found an uncertainty of 4.1 mm across the different Wii Balance Boards in static measurement; no information can be found in the literature about the reproducibility of measurement of center of pressure trajectories obtained with different Wii Balance Boards. However, this information is important to ensure repeatability and comparison of measurements between several clinical centers (eg, within multicenter studies organized to analyze a large amount of patients). This is an important question since this hardware was developed solely for gaming purposes and no scientific validation is available from the manufacturer.

The present study presents the repeatability of balance measurements using 4 different Wii Balance Boards systems.

## Methods

### Participants

Healthy adults (N=6) with a mean age of 36 years (SD 13), height of 176 cm (SD 11), and weight of 81 kg (SD 22), including 2 women participated in the study. This study was approved by the Ethical Committee of the Erasme Hospital (CCB B406201215142) and all participants provided informed consent.

### Measurement Setup

In order to assess the repeatability of Wii Balance Boards, the protocol of Huurnik et al was used [2]. A Wii Balance Board was placed on top of a force plate (AMTI model OR6-6, Watertown, MA, USA, size 50 cm × 46 cm) that was embedded within the laboratory floor. The sample rate for the force plate was 1000 Hz. Four different Wii Balance Boards were used (serial numbers BEH428405719, BEH428409281, BEH428408987, and BEH428409175). The Wii Balance Boards were connected to a laptop (Intel Core I5, Windows 7, 6 GB RAM) via Bluetooth connection, and data were retrieved using a custom-written software based on the Wiimotelib software [7]. The force plate was calibrated before the measurement was taken using the manufacturers’ recommendations. Although some methods have been proposed [1,2], no calibration procedure was used for the Wii Balance Board. Such calibration-free methodology was adopted because the purpose of this study was to evaluate the repeatability of measurements of the Wii Balance Board without the practical constraint of such systematic calibration procedures.

### Procedure

The participants performed 3 repetitions of double limb standing on each available Wii Balance Board in a single session; the 4 Wii Balance Boards were tested in this one session (12 trials per subject). Subjects were asked to stand in the middle of the Wii Balance Board for 30 seconds, as motionless as possible, eyes open, arms aligned along the body, and eyes fixed on a target on the wall in front of them. The same methodology was repeated for the 4 different Wii Balance Boards. The order of the tested Wii Balance Board system was randomly determined.

### Data Processing

**Figure 1:**
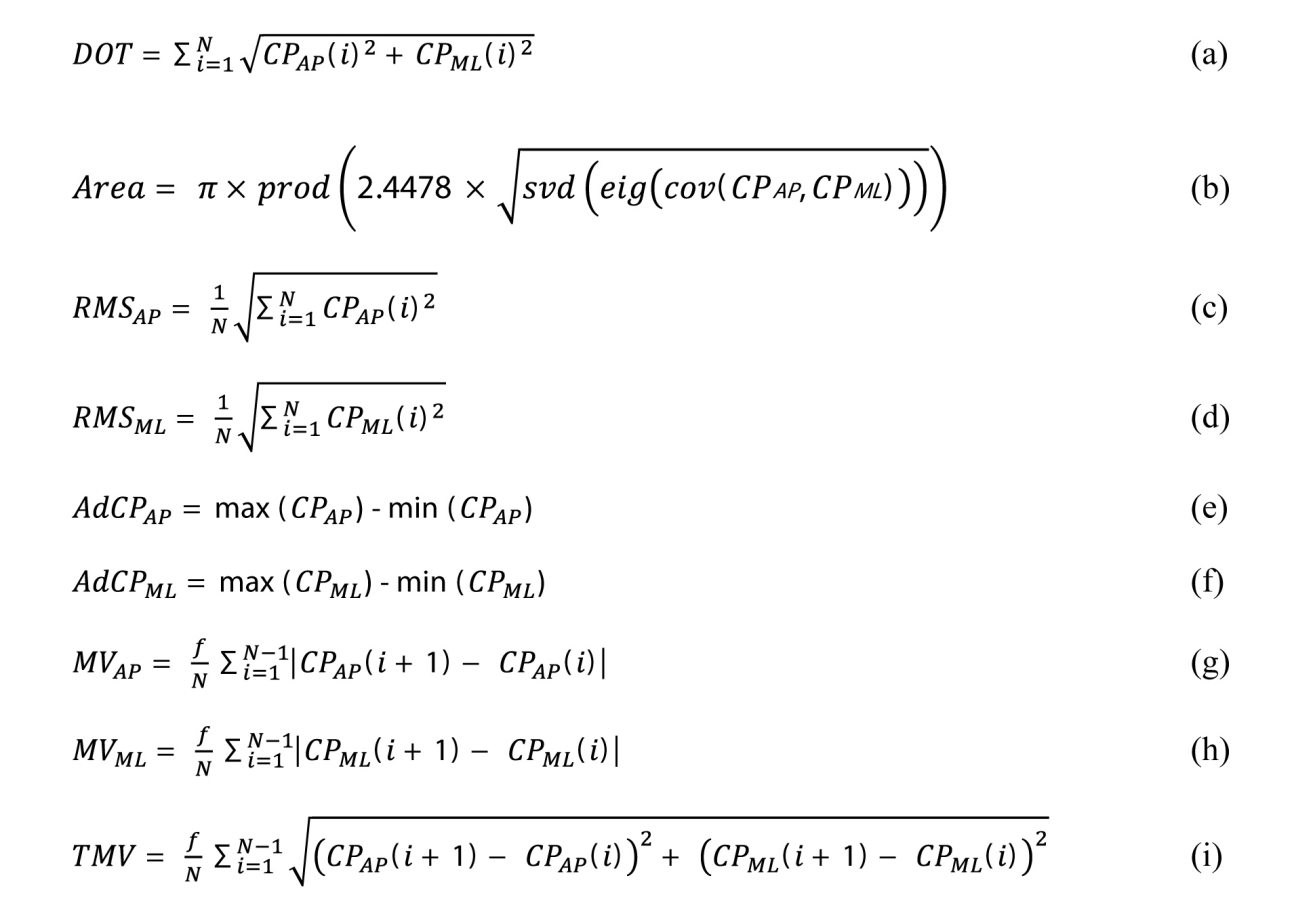
Equations.

Linear interpolation of the raw signals of the Wii Balance Board sensors was applied to get a regular sample rate of 1000 Hz (same as the force plate) [8]. Both data from the Wii Balance Board and force plate were then filtered using a second order Butterworth low-pass filter with a cutoff frequency of 12 Hz [2]. The displacements of the center of pressure along anterior-posterior (CP AP) and medio-lateral (CP ML) directions were obtained. Supplementary parameters were computed from the available center of pressure data using equations (Figure 1) based on previously published methodology [9]. The calculated parameters are defined in Textbox 1.

Since the data recorded during posture measurement was highly variable between trials (eg, various foot positions on the force plate and concentration of the subject) the mean difference between force plate and Wii Balance Board values was computed for each studied parameter, for each 30 second trial in order to tackle this variability. All statistics were performed on the mean of the 3 trials for each Wii Balance Board (6 subjects and 4 Wii Balance Boards each).

**Calculated parameters.**

Parameter

- DOT: total displacement of sway
- Area: the area of the 95% prediction ellipse (often referred to as the 95% confidence ellipse)
- SD AP and ML: the dispersion of center of pressure displacement from the mean position
- AdCP_AP_ and AdCP_ML_: the distance between the maximum and minimum center of pressure displacement
- MV_AP_ and MV_ML_: the mean velocity of center of pressure displacement
- TMV: the AP and ML displacements of the total center of pressure sway divided by the total duration of the trial

### Statistics

Intraclass correlation coefficients (ICC) (two-way random average measures; Model 2, single measurement) were computed to assess the reliability of the differences between devices and trials. Friedman tests (repeated measures) were also computed to compare the 4 different Wii Balance Boards. Differences between the force plate and Wii Balance Board for each trial (3 repetitions for each of the 4 Wii Balance Boards for each subject (N=6) totals 72 trials) with mean difference and confidence intervals plotted. The amount of observations that were outside the confidence intervals for each variable and each Wii Balance Board were summarized in a contingency table. Chi-square tests were computed to detect interactions between the Wii Balance Board and studied variables. Spearman correlation coefficients were computed between variables obtained from the force place and Wii Balance Board.

## Results

ICC results for agreement between Wii Balance Boards and results of the *F* and Friedman tests are presented in Table 1. Low agreements were found for variables derived from ML displacements (ICC values of .334 and .345 for RMS_ML_ and AdCP_ML_, respectively). Strong agreements were found for DOT (.704) and AdCP_AP_ (.786). Almost perfect agreements were found for the other variables (mean ICC=.962).

**Table 1:** Intraclass correlation coefficient (ICC) of the studied parameters for the 4 different devices.

Variable	ICC agreement	Bounds of the confidence interval		Friedman, *P* value
		Lower	Upper	
DOT	.704	-0.110	0.955	.93
Area	.959	0.858	0.994	.91
RMS_AP_	.906	0.686	0.985	.42
RMS_ML_	.334	-1.761	0.901	.87
AdCP_AP_	.786	0.336	0.965	.25
AdCP_ML_	.345	-1.911	0.904	.72
MV_AP_	.985	0.950	0.998	.95
MV_ML_	.980	0.931	0.997	.86
TMV	.984	0.945	0.997	.94

**Figure 2:**
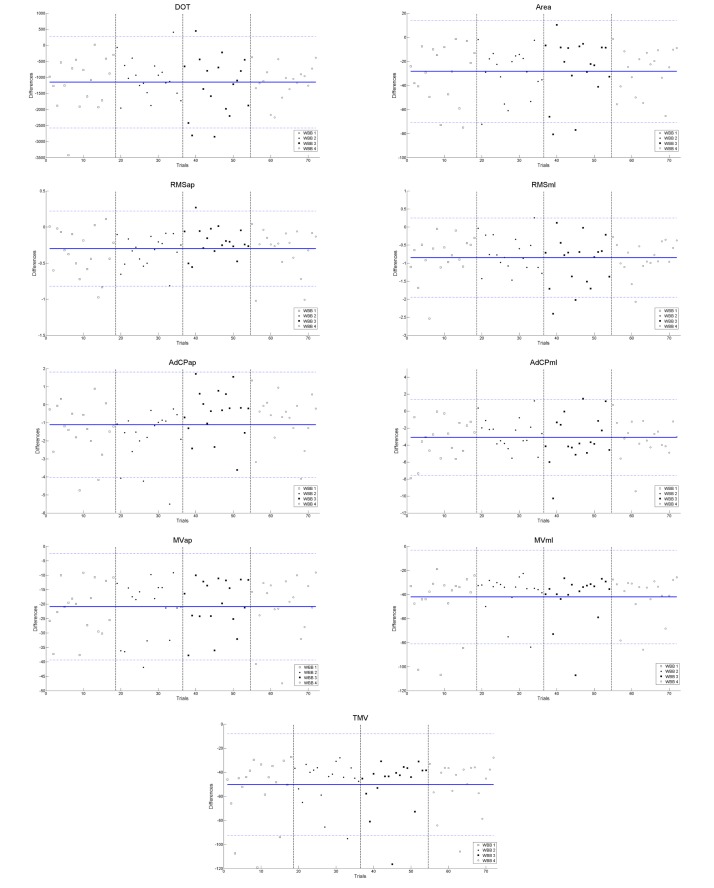
Differences between force plate and Wii Balance Boards for the 72 trials. Solid horizontal lines represent the mean difference. Dotted horizontal lines represent upper and lower confidence intervals (95%). Vertical lines indicate separation between the 4 different devices (18 trials per Wii Balance Board).

The differences between each trial and the mean difference with confidence intervals are presented in Figure 2. The contingency table of values that are outside the confidence intervals is presented in Table 2. The *P* value of the chi-square test is .59 (df=43); there is thus no association between the number of observations outside the confidence interval and the device.

The correlations between the data collected with the force plate and Wii Balance Boards are shown in Table 3. No statistical significant difference (Friedman test) was found for the correlations between the 4 different Wii Balance Boards.

**Table 2:** Contingency table of the number of observations outside of the confidence interval.

Variable	Wii Balance Board device number				
	1, n^a^	2, n^a^	3, n^a^	4, n^a^	Total, per variable
DOT	1 (0.05)	1 (0.05)	3 (0.16)	0 (0)	5 (0.07)
Area	2 (0.11)	1 (0.05)	2 (0.11)	0 (0)	5 (0.07)
RMS_AP_	2 (0.11)	0 (0)	1 (0.05)	2 (0.11)	5 (0.07)
RMS_ML_	1 (0.05)	0 (0)	2 (0.11)	1 (0.05)	4 (0.06)
AdCP_AP_	2 (0.11)	3 (0.16)	0 (0)	1 (0.05)	6 (0.08)
AdCP_ML_	1 (0.05)	0 (0)	2 (0.11)	1 (0.05)	4 (0.06)
MV_AP_	0 (0)	1 (0.05)	0 (0)	2 (0.11)	3 (0.04)
MV_ML_	3 (0.16)	1 (0.05)	1 (0.05)	1 (0.05)	6 (0.08)
TMV	3 (0.16)	1 (0.05)	1 (0.05)	1 (0.05)	6 (0.08)
Total, per device	15 (0.09)	8 (0.05)	12 (0.07)	9 (0.05)	44

^a^Values inside brackets represent the ratio between outside values and number of observations.

**Table 3:** Spearman correlation coefficients between the force plate and the 4 different Wii Balance Board devices.

Variables	Wii Balance Board device number				
	1, ρ	2, ρ	3, ρ	4, ρ	Mean, ρ
DOT	.79	.73	.73	.87	.78
Area	.85	.89	.82	.90	.86
RMS_AP_	.79	.92	.85	.78	.83
RMS_ML_	.85	.72	.72	.80	.76
AdCP_AP_	.76	.89	.74	.79	.80
AdCP_ML_	.89	.79	.76	.73	.79
MV_AP_	.94	.89	.88	.90	.91
MV_ML_	.96	.84	.85	.91	.89
TMV	.82	.80	.77	.86	.81
Mean, ρ (SD)	.85 (0.02)	.83 (0.02)	.79 (0.02)	.84 (0.02)	.83 (0.05)

## Discussion

### Principal Findings

To the best of our knowledge, this is the only study assessing the repeatability of measurements performed with different Wii Balance Boards. Our results confirm the findings of previous studies [1,2] where good correlations were found between Wii Balance Boards and force plates for center of pressure displacement. For other studied parameters derived from center of pressure displacement, good correlations were found.

With respect to the 4 different Wii Balance Boards, no differences were found between the boards (repeated measure Friedman test), but surprisingly, the ICC values showed low agreement for the ML parameters (RMS_ML_ and AdCP_ML_) although correlations with the force plate were high (.80 and.89 for RMS_ML_ and AdCP_ML_, respectively). The contingency table of the number of observations outside of the confidence intervals did not show particular errors for those measurements (Table 2). The other parameters had strong to almost perfect agreements. The fact that no differences were found between the different Wii Balance Boards (repeated measure Friedman test), and they all had good correlations to the force plate results indicates that different Wii Balance Boards can be used interchangeably. However, our results suggest that parameters derived from the ML displacement of center of pressure should be interpreted carefully.

This study presents results on several different parameters derived from center of pressure displacement. Most other studies have focused on investigating differences between Wii Balance Boards and a force plate for a single parameter (eg, center of pressure displacement) [1,2]. In addition to making a direct comparison over multiple boards with the same subjects and tasks, this study also compares multiple parameters.

### Limitations

The repeatability of some balance assessment protocols can be rather low. In our setup, we ran the risk of suffering from the same problem: in order to compare all the Wii Balance Boards and the force plate in a single trial, we would have had to place all the boards together on top of the force plate. As this is unfeasible, we processed our data by computing the difference between the Wii Balance Board and the force plate (for all the parameters) for each of the 4 trials. Over 3 trials and 6 subjects, we obtained 4 groups of 18 differences. ICCs were computed between those 4 groups. As the ICCs were high, the differences between the Wii Balance Board and force plate were considered consistent.

### Conclusions

This study indicates that balance and posture results recorded with one Wii Balance Board can be compared to the results recorded with another Wii Balance Board. This is particularly interesting for multicenter studies and supports the creation of larger populations for clinical studies. This study also allows us, in part, to address several criticisms that have recently been expressed. Some researchers [3,10] have doubts about the results of previous studies (past 3 years) on the validity of the Wii Balance Board. In addition to showing excellent correlation between the Wii Balance Board and force plate, this study shows that these high correlations are independent of the Wii Balance Board used.

## Abbreviations

AP: anterior-posterior
DOT: Total displacement of sway
ICC: Intraclass correlation coefficient
ML: medio-lateral
MV: Mean velocity
TMV: Total mean velocity
